# The association between asthma control, health care costs, and quality of life in France and Spain

**DOI:** 10.1186/1471-2466-13-15

**Published:** 2013-03-22

**Authors:** Marianne Doz, Christos Chouaid, Laure Com-Ruelle, Eduardo Calvo, Max Brosa, Julien Robert, Laurent Decuypère, Celine Pribil, Alicia Huerta, Bruno Detournay

**Affiliations:** 1 Cemka-Eval, 43 Boulevard du Maréchal Joffre, Bourg-la-Reine, F-92340, France; 2 APHP, Hôpital Saint-Antoine, Service de Pneumologie, 184 rue du Fbg Saint-Antoine, Paris, F-75571 cedex 12, France; 3 Institut de recherche et documentation en économie de la santé (IRDES), 10 rue Vauvenargues, Paris, F-75018, France; 4 Centro de Salud Universitario Pozuelo Estación, Madrid, Spain; 5 Oblikue Consulting, C/ Josep Irla i Bosh, 5-7, 1ª planta S-08034, Barcelona, Spain; 6 GlaxoSmithKline, 100 Route de Versailles, Marly-le-Roi cedex, France; 7 GlaxoSmithKline, Parque Tecnologico de Madrid, Severo ochoa 2, S-28760 Tres Cantos, Madrid, Spain

**Keywords:** Adult asthmatic patients, Asthma guidelines, EQ-5D-3L^®^, Drugs cost, Economic evaluation

## Abstract

**Background:**

Current asthma management guidelines are based on the level of asthma control. The impact of asthma control on health care resources and quality of life (QoL) is insufficiently studied. EUCOAST study was designed to describe costs and QoL in adult patients according to level of asthma control in France and Spain.

**Methods:**

An observational cost of illness study was conducted simultaneously in both countries among patients age greater or equal to 18 with a diagnosis of asthma for at least 12 months. Patients were recruited prospectively by GPs in 2010 in four waves to avoid a seasonal bias. Health care resources utilization of the three months before the inclusion was collected through physician questionnaires. Asthma control was evaluated using 2009 GINA criteria over a 3-month period. QoL was assessed using EQ-5D-3L^®^.

**Results:**

2,671 patients (France: 1,154; Spain: 1,517) were enrolled. Asthma was controlled in 40.6% [95% CI: 37.7% - 43.4%] and 29.9% [95% CI: 27.6% - 32.3%] of French and Spanish patients respectively.

For all types of costs, the percentage of patients using health care resources varied significantly according to the level of asthma control. The average cost (euros/3-months/patient) of controlled asthma was €85.4 (SD: 153.5) in France compared with €314.0 (SD: 2,160.4) for partially controlled asthma and €537.9 (SD: 2,355.7) for uncontrolled asthma (p<0.0001). In Spain, the corresponding figures were €152.6 (SD: 162.1), €241.2 (SD: 266.8), and €556.8 (SD: 762.4). EQ-5D-3L^®^ score was higher (p<0.0001) in patients with controlled asthma compared to partially controlled and uncontrolled asthma in both countries (respectively 0.88; 0.78; 0.63 in France and 0.89; 0.82; 0.69 in Spain).

**Conclusions:**

In both countries, patients presenting with uncontrolled asthma had a significantly higher asthma costs and lower scores of Qol compared to the others.

## Background

Asthma is a chronic disease with an estimated 300 million affected individuals throughout the world [1,2]. In a large French 2006 survey, 10.2% of a general population sample declared having suffered from asthma at least once in their life time and 6.7% had asthma at the time of the survey [3]. In Spain, the European Community Respiratory Health Survey (ECRHS-II) showed that the prevalence of asthma was 7% in some regional populations in 2007 [4].

The recent publication from the Global Initiative for Asthma (GINA) updated international guidelines and highlighted the importance of achieving and maintaining control as a goal of treatment [1,5]. Standardized tools [6-9] have been developed to assess asthma control.

Until now, some studies on asthma control based on data from large samples of asthmatic patients have already been published in Europe [10,11]. However, such studies were based on definitions of control that do not correspond to those of current GINA criteria. Moreover, asthma control was assessed over various periods of time (weeks/months) without taking into account symptoms seasonality [12].

Poor control of asthma may result in adverse clinical outcomes as well as substantial economic costs [13]. The total cost of asthma was estimated at 1.5 billion euros in France [14] and between 0.9 and 1.2 billion euros in Spain [15]. In 2006, the French ESPS survey estimated that the mean per-patient annual cost of ambulatory care was 1.6 times higher in partially controlled, and 2.9 times higher in uncontrolled asthma as compared to controlled asthma [16] but few European studies have provided data on costs related to asthma control. Finally, uncontrolled asthma may also have a negative impact on patient’s quality of life [17]. Data from the ECRHS-II showed that in patients with a known diagnosis of asthma, respiratory symptoms are important determinants of reduced health related quality of life (HRQL) [18]. Nevertheless, the specific relation between levels of asthma control and quality of life has been poorly documented [19-21].

Consequently, the EUCOAST (EUropean COst of ASthma Treatment) study was designed to assess utilisation of healthcare resources, costs and HRQL in adult patients with asthma in a real life setting in France and Spain accordingly to the level of asthma control.

## Methods

### Study design

An observational study was conducted in primary care settings in France and Spain in order to estimate the societal costs and the HRQL according to the level of asthma control in adult patients.

The study design required a single visit per patient. Data collection was performed on the three-month period before the inclusion. To take into account seasonality which has a major impact on asthma in both countries [12], patients were recruited during a period of one year in four quarterly waves from 01/01/2010 to 12/31/2010.

The EUCOAST study was approved by the French Consultative Committee for the data processing in health research (CCTIRS) and by the National commission for the personal data protection (CNIL). In Spain, the study obtained the authorisation of the Spanish Agency of Medicines and Medical Devices (AEMPS) and the favourable opinion of the Clinical research Ethics Committee (CEIC) Hospital Clínico y Provincial de Barcelona.

### Populations

Patients were included if they met the following inclusion criteria: adults aged 18 or over, diagnosed with asthma for at least 12 months and having received at least one anti-asthmatic treatment (whatever the treatment) within the past 12 months.

Exclusion criteria were participation into a clinical trial during the past 6 months, aged 45 years and over with a history of smoking of at least 20 pack years, chronic obstructive pulmonary disease (COPD) or pregnancy.

Based on the percentage of patients with controlled asthma estimated to represent 40% to 50% of the overall population in previous studies [22], and knowing that the size of the sample needed to estimate percentage with a ± 5% value according to the confidence interval selected (alpha risk 5%, normal distribution), around 380 patients had to be enrolled quarterly.

In France, investigators were a sample of GPs selected from a representative panel of 1,200 general practitioners. The panel's representativeness was established by three criteria: age, sex and region of practice. The study was proposed to 750 GPs of this panel, randomly selected and 230 agreed to participate.

In Spain, 105 general practitioners of 18 autonomous communities belonging to 3 regions (North, South and Mediterranean) were contacted and 87 agreed to participate. A territorial representativeness was able to be obtained when the sample was constituted.

Practitioners had to include patients in the usual context of their practice. To avoid a selection bias, the first two consecutive patients (the first five in Spain) corresponding to the inclusion/exclusion criteria visiting their doctor were included in each wave.

### Data collection

Data collected retrospectively (over the last 3 months) during the single study visit by the GP were: socio-demographic characteristics, medical history of patients, GINA criteria (including occurrences of exacerbations, limitations of activities, daytime and nocturnal symptoms, need of rescue/reliever treatment) and healthcare resource use due to asthma.

For all medical resources consumption (including sick leaves) during the 3 months prior to GP visit, data were provided by GPs through the questionnaires used in the study. In France some data (i.e. prescription drugs related to asthma were also collected through the computerized medical files of the GPs).

Force Expiratory Volume in one second (FEV1) was collected only in a single visit (FEV1 was measured by GPs three times consecutively as recommended by GINA). As in France and Spain, all investigators were given a digital device (Mini-Wright™ Digital, Clement Clarke International Ltd) which allowed FEV1 measurements.

At the time of the inclusion, patients completed the EQ-5D-3L^®^ questionnaire comprising a Visual Analog Scale (VAS). This questionnaire is a generic instrument [23] which comprises 5 dimensions: mobility, self-care, usual activity, pain/discomfort and anxiety/depression and it is widely used to assess HRQL in asthma patients [24,25]. EQ-5D-3L^®^ health states were valuated using validated French [26] and Spanish [27] utility value set available in each country.

### Data analysis

In France, data were weighted to compensate the disproportion of inclusions observed among the different quarterly waves. Individual case weights were defined according to the ratio between the number of inclusions in wave 1 and the number of inclusions in the subsequent waves. In Spain, as the number of inclusions was similar in each wave, it was not necessary to weight the data.

Data analysis was performed for patients for whom lung function and symptoms of asthma were duly collected in the medical questionnaire. According to GINA 2009 criteria, patients were classified in 3 subgroups (“controlled, “partially controlled” and “uncontrolled” patients); statistic tests were performed to compare these 3 subgroups.

For categorical variables (age, gender, co-morbidities, exacerbations, smoking status, FEV1, visits, hospitalization, ambulatory exams, emergency room, drugs, sick leave), Pearson’s Chi2 test or Fisher’s Exact Test were applied. For continuous variables (costs, EQ-5D-3L^®^ scores, VAS score, and number of years with a diagnosed asthma), analysis of the variance was performed. If the data were not normally distributed, non-parametric tests were used. The cost analysis was carried out according to a societal perspective and took into account both direct and indirect costs (costs associated with sick leave). Considering differences between the Spanish and the French healthcare systems, it was judged as not relevant to compare cost data across the two countries. A unit cost was given to each item according to the tariff currently used in 2010^a,b^. A weighted average cost was then calculated. In France, accordingly to national guidelines, productivity loss was estimated by using the human capital method. The Gross Domestic Product for the year 2008 was divided by the number of employed population given by the “Institut National de la Statistique et des études économiques” (INSEE) for the same year. Then, the GDP per capita (employed population only) was divided by the number of working days. In Spain the average annual salary was used and sick leaves were valued by using the average annual salary divided by the number of working hours in a year (provided in Instituto Nacional de Estadística). In both case, results were multiplied by the duration of sick leaves collected through the survey.

As data were collected on a 3-month period, cost data were not discounted.

Multivariate regression analyses were performed to examine the relation between level of asthma control and the outcomes (direct costs only or HRQL levels). The costs or HRQL levels were the dependent variable and control level was the independent (explanatory) variable.

The Tobit model was used for the relation between cost and level of asthma control in order to take into account patients with zero costs (i.e., left-censoring of cost data). Multiple linear regression was used to estimate the effect associated with level of control on HRQL. The potentially confounding factors taken into account in the models were sex, age, episodes of exacerbation of asthma, prescription of a controller treatment and follow-up by a lung specialist.

## Results

### Study population

**Figure 1:**
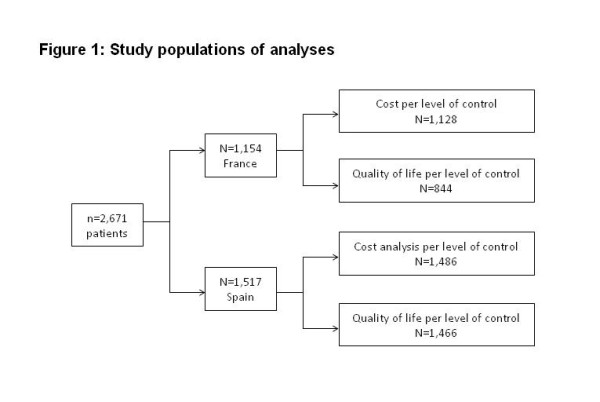
Study populations of analyses.

Two hundred thirty-eight investigators (155 in France and 83 in Spain) enrolled 2,671 patients (1,154 in France and 1,517 in Spain), Data on FEV1 or asthma symptoms were missing for 26 patients in France and 31 in Spain), and data on HRQL were missing for 310 patients in France and 51 in Spain (Figure 1). Table 1 presents characteristics of the study population by country and according to the level of control.

**Table 1:** Characteristics of the asthmatic population in France and Spain according to the level of control

**Over a 3-month period**	**France 1,154 (100%)**				**Spain 1,517 (100%)**			
**Level of asthma control**	**Controlled 458 (40.6%)**	**Partially controlled 428 (38.0%)**	**Uncontrolled 242 (21.4%)**	**p**	**Controlled 445 (29.9%)**	**Partially controlled 506 (34.1%)**	**Uncontrolled 535 (36.0%)**	**p**
Gender				0.008				0.3337
Male	45.1%	37.8%	33.9%		38.7%	37.2%	34.2%	
Female	54.9%	62.2%	66.1%		61.3%	62.8%	65.8%	
Age				0.0132				<0.0001
[18-45[	40.2%	42.7%	35.6%		60.3%	52.2%	44.8%	
[45-65[	42.2%	36.5%	36.3%		27.7%	25.9%	35.3%	
≥65	17.6%	20.7%	28.2%		12.0%	21.9%	19.9%	
Mean (SD)	48.4 (16.6)	49.0 (18.0)	53.3 (18.3)	0.0013	42.4 (15.9)	45.6 (19.0)	47.8 (17.7)	<0.0001
Number of years with a diagnosed asthma								
Mean (SD)	17.8 (13.7)	19.1 (14.1)	20.9 (14.6)	0.0234	13.2 (10.5)	13.7 (10.4)	15.8 (11.7)	0.0004
Comorbidities (at least one)	68.8%	73.1%	79.0%	0.0154	72.1%	76.5%	83.1%	0.0002
Allergic rhinitis	70.2%	68.8%	62.4%	0.1668	81.0%	77.0%	70.9%	0.0047
Atopic dermatitis	13.6%	14.2%	19.2%	0.1983	17.4%	18.9%	24.5%	0.0325
Others allergies	15.1%	16.3%	12.0%	0.4036	12.3%	11.1%	13.3%	0.6355
GERD Ŧ	18.9%	24.6%	25.9%	0.1099	12.3%	9.8%	19.4%	0.0002
Depression	12.3%	17.8%	30.0%	<0.0001	7.9%	12.7%	16.7%	0.0017
Another disease related to asthma	6.5%	10.0%	13.0%	0.0453	8.7%	11.1%	9.0%	0.4783
Asthma exacerbations (last 3 months) Yes	18.5%	36.2%	62.8%	<0.0001	9.2%	41.3%	74.4%	<0.0001
Smoking status				0.1076				0.0461
Current smoker	14.0%	20.0%	18.7%		13.0%	16.7%	18.8%	
Former smoker	17.6%	13.6%	15.4%		18.0%	12.9%	15.4%	
Never smoker	68.4%	66.4%	65.9%		69.0%	70.4%	65.9%	
Lung function (FEV1∞)				<0.0001				<0.0001
<50%	4.7%	8.3%	15.5%		0.9%	5.0%	5.8%	
50-79%	10.6%	23.9%	27.3%		8.4%	20.7%	22.1%	
≥80%	84.8%	67.8%	57.2%		90.7%	74.4%	72.0%	

Ŧ Gastroesophageal reflux disease.

∞ Forced expiratory volume in one second.

#### France

Asthma was considered as controlled over the last 3 months in 40.6% [95% CI: 37.7% - 43.4%], partially controlled in 38.0% [95% CI: 35.2% - 40.8%] and uncontrolled in 21.4% [95% CI: 19.1% - 23.8%] of patients.

Among patients with an uncontrolled asthma a higher percentage of women (66.1% for *vs*. 62.2% for partially controlled *vs.* 54.9% for controlled, p<0.0001) and patients aged 65 and over (28.2% *vs*. 20.7% for partially controlled *vs.* 17.6% for controlled, p<0.0001) were observed.

Those with uncontrolled asthma had a higher rate of depression (p<0.0001) than patients with partially controlled or controlled asthma. Prevalence of allergic rhinitis, atopic dermatitis, or gastroesophageal reflux was not significantly related to asthma control.

Asthma exacerbations occurred for 34.6% of patients during the last 3 months with an average number of 2.3 (SD: 3.0) episodes/patient with exacerbations/quarter. As expected, the percentage of patients who had at least one exacerbation was significantly higher (p<0.0001) in patients with uncontrolled asthma (62.8%) compare to partially controlled patients (36.2%) and controlled patients (18.5%). Overall, 70.4% of patients had a normal lung function (FEV1 ≥80%): 84.8% of those with controlled asthma *vs.* 67.8% for partially controlled and 57.2% for uncontrolled asthma (p<0.0001).

#### Spain

The proportion of patients with controlled, partially controlled and uncontrolled asthma were 29.9% [95% CI: 27.6% - 32.3%], 34.1% [95% CI: 31.6% - 36.5%] and 36.0% [95% CI: 33.6%-38.5%], respectively. The level of asthma control was lower in Spain than in France (p<0.001). Patients with controlled asthma were younger than patients with uncontrolled asthma (p<0.0001).

There was a significant relation between asthma control and rate of allergic rhinitis, atopic dermatitis, and gastroesophageal reflux as well as depression with more frequent comorbidities in patients with uncontrolled asthma. 43.6% of patients suffered from asthma exacerbations during the last 3 months with an average number of 1.8 (SD1.7) episodes/patient with exacerbations/quarter: 74.4% of those with uncontrolled asthma, 41.3% with partially controlled and 9.2% with controlled asthma.

Overall, 78.7% of patients had a normal lung function (90.7% of those with controlled *vs.* 74.4% with partially controlled *vs.* 72.0% with uncontrolled asthma).

### Health resources consumptions and cost of asthma

#### France

Detailed analyses showed that, for all types of costs, the percentage of patients with medical resource consumption varied significantly according to the level of asthma control (Table 2).

**Table 2:** Asthma associated consumption of medical resources within the last 3 months according to the level of control

**Over a 3-month period**	**France 1,154 (100.0%)**				**Spain 1,517 (100.0%)**			
**Asthma control**	**Controlled 458 (40.6%)**	**Partially controlled 428 (38.0%)**	**Uncontrolled 242 (21.4%)**		**Controlled 445 (29.9%)**	**Partially controlled 506 (34.1%)**	**Uncontrolled 535 (36.0%)**	
	**%**	**%**	**%**	**p**	**%**	**%**	**%**	**p**
At least one prescription of antiasthmatic drugs	57.9	67.3	71.7	0.0004	78.4	87.7	92.9	<0.0001
*Reliever treatment only*	*6.9*	*6.8*	*5.9*	0.8583	*18.2*	*16.2*	*10.8*	0.0032
*Controller or Reliever and controller treatment*	*51*	*60.5*	*65.8*	0.0003	*60.2*	*71.5*	*82.1*	<0.0001
At least one GP Visit	35.4	44.5	70.0	<0.0001	47.2	64.2	81.9	<0.0001
At least one specialist visit	2.1	5.1	15.7	<0.0001	11.2	13.8	22.2	<0.0001
At least one physiotherapist visit	0.2	0.3	3.1	<0.0001	1.1	1.2	4.3	0.0005
At least one nurse visit	0.2	0.4	3.1	<0.0001	5.8	9.7	17.8	<0.0001
At least one emergency room visit	0	0.7	3.6	<.0001	0.7	2.4	14.4	<0.0001
At least one hospitalization	0	0	2.1	NA	0	1.0	7.3	<0.0001
At least one ambulatory exam	2.1	5.6	9.7	<0.0001	27.0	27.5	38.3	<0.0001
At least one sick leave	0.5	2.1	5.3	0.0002	0.7	1.6	13.9	<0.0001

*NA* Not applicable.

The average per-patient total cost of asthma-related healthcare was €537.9 in uncontrolled patients, €314.0 in partially controlled patients and €85.4 in controlled patients (p<0.0001). Antiasthmatic drugs represented the main driver of direct costs, 86.2%, 81.9% and 61.5% in controlled, partially controlled and uncontrolled patients, respectively.

Indirect costs were marginal in controlled patients (4.9% of the total costs) but represented a major driver in partially controlled and uncontrolled patients (respectively 62.8% and 58.1%) (Table 3).

**Table 3:** Mean per-patient asthma-related total cost (euros), societal perspective, according to the level of control GINA (last 3 months)

	**Societal perspective, France**				**Societal perspective, Spain**			
**Asthma control**	**Controlled N=458**	**Partially controlled N=428**	**Uncontrolled N=242**		**Controlled N=445**	**Partially controlled N=506**	**Uncontrolled N=*535***	
	**Mean (sd)**	**Mean (sd)**	**Mean (sd)**	p*	**Mean (sd)**	**Mean (sd)**	**Mean (sd)**	p*
Antiasthmatic drugs (€)	70.0 (108.3)	95.7 (141.2)	138.6 (231.4)	<0.0001	87.7 (103.9)	121.1 (121.2)	158.4 (141.3)	<0.0001
*Reliever treatment only*	*4.5 (16.6)*	*8.4 (25.3)*	*16.4 (49.4)*	*<0.0001*	*3.6 (13.8)*	*5.6 (21.8)*	*13.4 (37.3)*	*<0.0001*
*Controller treatment*	*23.0 (61.5)*	*32.3 (68.6)*	*50.0 (112.0)*	*0.0011*	*24.6 (60.1)*	*32.5 (65.1)*	*41.6 (67.9)*	*<0.0001*
*Fixed association*	*42.5 (78.4)*	*55.0 (97.1)*	*72.2 (118.8)*	*0.0005*	*59.5 (77.6)*	*83.0 (88.4)*	*103.4 (95.9)*	*<0.0001*
GP Visits (€)	9.7 (15.6)	15.8 (26.2)	36.4 (50.6)	<0.0001	35.3 (57.7)	71.4 (90.1)	142.9 (148.8)	<0.0001
Specialist visits	0.5 (3.6)	2.0 (10.2)	5.7 (14.8)	<0.0001	7.5 (22.4)	12.2 (37.5)	23.0 (56.8)	<0.0001
Physiotherapist visits (€)	0.0 (0.6)	0.1 (1.3)	2.5 (19.8)	<0.0001	0.3 (2.8)	0.2 (2.2)	1.0 (5.5)	0.0005
Nurse visits (€)	0.0 (0.2)	0.0 (0.5)	0.5 (4.5)	<0.0001	1.9 (9.8)	3.2 (12.2)	7.7 (22.5)	<0.0001
Emergency room (€)	0.0 (-)	0.9 (11.5)	4.0 (22.1)	<0.0001	0.9 (11.3)	4.1 (29.2)	25.3 (69.2)	<0.0001
Hospitalization (€)	0.0 (-)	0.0 (-)	33.6 (227.1)	<0.0001	0.0 (-)	3.9 (44.6)	55.3 (268.5)	<0.0001
Ambulatory exams (€)	1.0 (7.3)	2.4 (10.5)	4.0 (13.2)	0.0002	15.0 (32.3)	13.9 (29.9)	20.8 (36.7)	0.0001
**TOTAL DIRECT COST (€)**	**81.2 (113.3)**	**116.9 (155.4)**	**225.3 (352.1)**	**<0.0001**	**148.7 (147.3)**	**229.9 (217.3)**	**434.4 (497.7)**	**<0.0001**
Sick leaves (indirect costs) (€)	4.2 (83.0)	197.1 (2,125.8)	312.6 (2,286.2)	0.0002	3.9 (56.3)	11.3 (109.5)	122.4 (410.7)	<0.0001
**TOTAL COST (€)**	**85.4 (153.5)**	**314.0 (2,160.4)**	**537.9 (2,355.7)**	**<0.0001**	**152.6 (162.1)**	**241.2 (266.8)**	**556.8 (762.4)**	**<0.0001**

*The tests were performed to compare controlled, partially controlled and uncontrolled patients.

The multivariate analysis showed that total direct costs over 3 months were higher in patients with uncontrolled asthma rather than in patients with controlled asthma (+€112.8 in uncontrolled patients *vs*. controlled), older people (≥65) rather than younger ones patients (+€49.), experiencing exacerbations (+€75.5), treated with controller treatment (+€253.7, or visiting a lung specialist (+€62.4), (Table 4). Such figures must be interpreted with caution due to the fact that the level of control may be the consequence of other covariables (i.e. the controller treatment).

**Table 4:** Multivariate analyses of asthma related direct costs (€) (3-month observational period)

***Country***		**France**		**Spain**	
**Direct asthma related costs**		**Estimated Value**	**CI 95%**	**Estimated Value**	**CI 95%**
Level of control measured with GINA	Partially controlled (*vs.* controlled)	14.6	[-15.1 ; 44.3]	1.4	[-40.1 ; 42.9]
	Uncontrolled (*vs.* controlled)	110.9*	[74.9 ; 146.8]	104.6*	[58.1 ; 151.1]
Gender	Men *vs.* women	−19.5	[-45.6; 6.6]	50.9*	[18.2 ; 83.6]
Age	45-64 years (*vs.* ≥ 18-44years)	39.2*	[10.1 ; 68.3]	88.3*	[51.5 ; 125.1]
	≥ 65 years (*vs.* ≥ 18-44years)	51.3*	[17.1 ; 85.5]	79.6*	[35.3 ; 123.9]
Presence of exacerbation	Yes (*vs.* None)	74.6*	[46.5 ; 102.6]	159.8*	[122.4 ; 197.2]
Patient with at least one co-morbidity**	Yes (*vs.* None)	26.8	[-2.3 ; 55.9]	11.8	[-26.3 ;49.8]
Controller treatment	Yes (*vs.* None)	254.3*	[226.9 ; 281.6]	221.6*	[184.4 ; 258.7]
Patient followed by a lung specialist	Yes (*vs.* None)	59.7*	[33.7 ; 85.6]	218.7*	[181.0 ; 255.3]

* p<0.05.

** In France, the most frequent co-morbidity was depression and in Spain it was gastroesophageal reflux.

#### Spain

The mean per-patient total cost of asthma-related healthcare was €556.8 in uncontrolled patients, €241.2 in partially controlled patients and €152.6 in controlled patients (p<0.0001) (Table 3). 92.9% of patients with uncontrolled, as compared with 78.4% of patients with controlled asthma, were prescribed at least one antiasthmatic drugs during the observation period (p<0.05) (Table 2).

Antiasthmatic medications represented the main driver of direct costs for controlled and partially controlled asthma (respectively 59.0% and 52.7%) but not for uncontrolled asthma (36.5%). For the latter GPs visits had also a major impact on costs accounting for 32.9% of the mean per-patient direct costs.

Indirect costs were marginal in controlled and in partially controlled patients (respectively 2.6% and 4.7% of the total costs) but much more important (22%) in uncontrolled patients.

In multivariable analyses, factors predicting asthma costs were the same in both countries except for gender which had a significant impact in Spain but not in France (Table 4).

### Quality of life in France and Spain

In both countries (Table 5), average EQ-5D-3L^®^ quality of life scores were higher for patients with controlled asthma than patients with partially controlled or uncontrolled asthma (0.88 *vs*. 0.78 *vs*. 0.63 in France and 0.89 *vs*. 0.82 *vs*. 0.69 in Spain; p<0.0001).

**Table 5:** EQ-5D-3L^®^ scores per level of control

			**Quality of life according to GINA (last 3 months)**			
			**Controlled**	**Partially controlled**	**Uncontrolled**	**p**
EQ-5D-3L^^®^^ score	France	N	344	307	182	
		Mean (SD)	0.88 (0.18)	0.78 (0.23)	0.63 (0.28)	<0.0001
	Spain	N	436	498	529	
		Mean (SD)	0.89 (0.16)	0.82 (0.20)	0.69 (0.24)	<0.0001
VAS	France	N	332	297	171	
		Mean (SD)	77.33 (15.02)	70.27 (16.74)	57.41 (18.30)	<0.0001
	Spain	N	435	496	528	
		Mean (SD)	80.00 (14.13)	75.13 (16.17)	62.81 (18.11)	<0.0001

Detailed analyses of EQ-5D-3L^®^ results per dimension (mobility, autonomy, daily activities, pain/discomfort, and anxiety/depression) showed consistent differences between controlled, partially controlled and uncontrolled patients. For all dimensions, quality of life scores were better for controlled patients (p<0.0001) (data not shown).

The Visual Analog Scale (VAS) quality of life scores were also significantly associated with the level of asthma control in both countries (p-values < 0.001). Average VAS scores were 77.33, 70.27, 57.41 in France and 80.00, 75.13, 62.81 in Spain for controlled, partially controlled and uncontrolled patients, respectively (Table 5).

In France, using multi-variable regression analyses, the EQ-5D-3L^®^ scores (Table 6) were significantly lower for patients having an uncontrolled (estimated value -0.22) or partially controlled asthma (estimated value -0.09) as compared with controlled asthma. They were also lower for female patients and patients aged 65 years or more (estimated value -0.13) or 45-64 (estimated value -0.07) rather than those aged 18-44 years.

**Table 6:** Multivariate analyses of EQ-5D-3L^®^ utility score (3-month observational period)

***Country***		**France**		**Spain**	
**EQ-5D-3L^^®^^ 3L utility score**		**Estimated Value**	**CI 95%**	**Estimated Value**	**CI 95%**
Level of control measured with GINA	Partially controlled (*vs.* controlled)	−0.093*	[-0.128 ; -0.058]	−0.042*	[-0.068 ; -0.017]
	Uncontrolled (*vs.* controlled)	−0.220*	[-0.263 ; -0.177]	−0.158*	[-0.186 ; -0.129]
Gender	Men *vs.* women	0.056*	[0.019 ; 0.081]	0.052*	[0.032 ; 0.072]
Age	45-64 years (*vs.* ≥ 18-44years)	−0.071*	[-0.105 ; -0.036]	−0.101*	[-0.123 ; -0.078]
	≥ 65 years (*vs.* ≥ 18-44years)	−0.131*	[-0.171 ; -0.091]	−0.175*	[-0.202 ; -0.148]
Presence of exacerbation	Yes (*vs.* None)	−0.011	[-0.044 ; 0.024]	−0.005	[-0.028 ; 0.017]
Patient with at least one co-morbidity	Yes (*vs.* None)	−0.019	[-0.053 ; 0.014]	−0.028*	[-0.051 ; -0.004]
Controller treatment	Yes (*vs.* None)	−0.025	[-0.056 ; 0.006]	0.009	[-0.012 ; 0.032]
Patient followed by a lung specialist	Yes (*vs.* None)	−0.016	[-0.046 ; 0.015]	−0.037*	[-0.059 ; -0.014]
**VAS Score**		**Estimated Value**	**CI 95%**	**Estimated Value**	**CI 95%**
Level of control measured with GINA	Partially controlled (*vs.* controlled)	−5.777*	[-8.331 ; -3.223]	−2.762*	[-4.850 ; -0.675]
	Uncontrolled (*vs.* controlled)	−16.758*	[-19.959 ; -13.556]	−13.255*	[-15.609 ; -10.901]
Gender	Men *vs.* women	3.456*	[1.198 ; 5.714]	2.232*	[0.577 ; 3.887]
Age	45-64 years(*vs.* ≥ 18-44years)	−5.966*	[-8.480 ; -3.453]	−8.492*	[-10.359 ; -6.625]
	≥ 65 years (*vs.* ≥ 18-44years)	−11.179*	[-14.140 ; -8.218]	−12.258*	[-14.507 ; -10.008]
Presence of exacerbation	Yes (*vs.* None)	−2.542*	[-5.077 ; -0.007]	−2.967*	[-4.872 ; -1.063]
Patient with at least one co-morbidity	Yes (*vs.* None)	−3.283*	[-5.735 ; -0.831]	0.092	[-1.842 ; 2.027]
Controller treatment	Yes (*vs.* None)	−0.749	[-3.037 ; 1.539]	1.788	[-0.064 ; 3.642]
Patient followed by a lung specialist	Yes (*vs.* None)	−1.721	[-3.955 ; 0.513]	−3.085*	[-4.981 ; -1.188]

* p<0.05.

Similar results were observed in Spain. In addition, in that country, follow-up by a pulmonary specialist was also significantly associated with a decrease in quality of life (estimated value -0.06; p<0.0003).

Results of the multivariable analysis using the VAS score as the dependent variable were consistent with those reported above for the EQ-5D-3L^®^. In addition, exacerbations were significantly associated with a lower VAS score in both countries.

## Discussion

In this observational study we found that the proportion of patients with controlled asthma was significantly higher in France (41%) than in Spain (30%). In both countries, costs were higher and HRQL lower as level of asthma control decreased. The average asthma-related total health care costs over a three-month period were €85.4, €314.4 and €537.9 in France and €152.6, €241.2 and €556.8 in Spain for patients with controlled, partially controlled and uncontrolled asthma, respectively. The HRQL scores (EQ-5D-3L^®^) were 0.88, 0.78 and 0.63 in France (p<0.0001) and 0.89, 0.82 and 0.69 (p<0.0001) in Spain, for patients with controlled, partially controlled and uncontrolled asthma, respectively. According to the GINA 2009 Guidelines [1], the goal of asthma treatment is to achieve and maintain asthma control. The level of control is assessed based on symptoms, the use of reliever treatments, the adaptation of daily life, and measurement of peak expiratory flow or FEV1. However, this assessment of the level of asthma control may be conducted in different ways and the level of control can also vary over time.

The GINA 2009 criteria did not specify precisely the period over which asthma control should be assessed. Instead, any of the criteria observed over a given week may affect the level of asthma control for the whole period. Recently, for the first time, GINA 2010 [28] guidelines provided a time frame for the assessment of asthma control and recommended that asthma control must be assessed “preferably over 4 weeks”. In our study, asthma control was assessed using symptoms data on a three-month period but FEV1 was measured only at the end of that period of time and this may be considered as a limitation of our study.

Previous studies estimated that approximately 40% of patients in France [29] have uncontrolled asthma. Similar figures were observed in Spain [30]. These estimates are higher than those in our study in France (uncontrolled asthma=21.4%) but consistent with our results in Spain (uncontrolled asthma=36%). However, the differences between our results and previous ones may be the consequence of slightly different definitions of asthma control. The reasons for the differences in asthma control between the two neighbouring countries are not known. Possible explanations include differences in patient compliance with asthma treatments, as well as environmental and genetic factors. In addition, there may be measurement issues as physicians may vary in their assessment of GINA’s criteria.

Indeed, an important study found also great variability in the prevalence of uncontrolled asthma across European countries [10].

In our study, the average total cost per patient over a 3-month period was higher when asthma was poorly controlled. Hospitalizations for asthma and emergency room visits associated costs were higher in patients with uncontrolled asthma and they represent a higher percentage of the total direct costs (16.7% and 18.6% of the total direct cost in France and Spain respectively *versus* 0% and 0.6% in patients with controlled asthma). This result is in line with previous European results [14,16,21].

However, antiasthmatic drugs were the main driver of direct costs in both countries. In addition, the use of controller treatment was associated with the highest direct asthma-related costs regardless of the level of control and after adjustment for several potentially confounding factors (except in the subgroup of Spanish patients with uncontrolled asthma).

The EUCOAST study presents several limits. First, both the sampling frame and the sampling method used did not ensure the representativeness of the investigators even if their main characteristics were comparable to those of all French and Spanish GPs.

Secondly, as investigators had to recruit several patients (until 5 in Spain) there was a potential within-unit correlation in the data which was not taken into account in the statistical analysis.

Thirdly, due to a possible short term memory bias GPs and patients may have under-reported healthcare consumptions. Therefore, costs may be lower than those found in studies based on claims databases or systematic healthcare data. However, it is difficult to know whether, or to what extent, this might have biased our estimates of the effects associated with level of asthma control. Indeed, the existence of such bias would depend on whether under-reporting of costs was differential according to level of asthma control.

Costs of medications were based on prescription data. As some patients may not have been compliant, our estimates may be over-estimates of the true medication-related costs.

The response rate for the EQ-5D-3L^®^ was lower in France as compare to Spain (73% *vs.* 97%). This was likely the consequence of differences in the HRQL data collection method in the two countries. In France, patients were asked to send back their questionnaires to the study center whereas in Spain most patients completed the questionnaire in the physician’s office. However, we did not find significant differences between responders and non-responders in their level of asthma control or overall costs.

## Conclusion

Asthma control was significantly associated with costs and health-related quality of life in both France and Spain. Despite differences in health care systems and after adjustment for several potentially confounding factors, in both countries, costs were higher and HRQL lower as level of asthma control decreased.

These results suggest that implementation of measures to improve asthma control may improve patients’ quality of life and reduce related costs for National Health Systems and for the society.

### Endnotes

^a^In France: public prices inclusive of tax were used to get the unit cost of drugs; the rates for all medical and paramedical procedures were established from national conventions and from the Common Classification of Medical Procedures for the year remove one as it is written twice 2010.

^b^In Spain, the database used for drug costing was that of the Consejo General de Colegios Oficiales de Farmacéuticos (http://www.portalfarma.com). For other resources, costing was carried out using a national Spanish base (Spanish Health costs database eSalud).

## Abbreviations

AEMPS: Spanish Agency of Medicines and Medical Devices, CCTIRS, Consultative Committee for the data processing in health research; CEIC: Clinical research Ethics Committee; CI: Confidence interval; CNIL: National commission for the personal data protection COPD, chronic obstructive pulmonary disease, CSD, Cegedim Strategic Data; ECRHS-II: European Community Respiratory Health Survey-II; ESPS: Enquête Santé Protection Sociale; EUCOAST: EUropean COst of ASthma Treatment; FEV1: Force expiratory volume in one second; GERD: Gastroesophageal reflux disease; GINA: Global initiative for asthma; GPs: General practitioners; HRQL: Health related quality of life; QoL: Quality of life; SD: Standard deviation; SRAP: Sociedad de Respiratorio de Atención Primaria; VAS: Visual analog scale

## Competing interests

Doz M, Robert J and Detournay B are consultants for CEMKA-EVAL, a company providing consultancy services for most pharmaceutical companies and public institutions involved in health care in France. Chouaid C has received consulting fees or honoraria from Novartis, Boehringer Ingelheim, AstraZeneca and GlaxoSmithKline. Calvo E has worked as national coordinator for primary care in international clinical trials sponsored by Astra Zeneca and has been principal or secondary investigator in national studies conducted by Astra Zeneca, Boehringher, MSD, GlaxoSmithKline and Abbott. He has also written papers or given courses and conferences sponsored by Boehringher, Pfizer, AstraZeneca, GlaxoSmithKline, Chiesi, Merck and Almirall. He has participated in advisory committees for GlaxoSmithKline, Nycomed and Novartis. Com-Ruelle L received consulting fees or honoraria from GlaxoSmithKline as an expert of the study scientific committee. Brosa M is consultant for Oblikue, a company providing consultancy services for most pharmaceutical companies and public institutions involved in health care in Spain. Decuypère L and Pribil C are employed at GlaxoSmithKline France. Huerta A is employed at GlaxoSmithKline Spain.

## Authors’ contribution

DM coordinated the data collection and wrote the manuscript. CC, CRL, CE, BM and DL participated in the interpretation of data, were involved in drafting the manuscript and revised it critically for important intellectual content. RJ performed all statistical analysis. PC and HA participated in study conception and interpretation of data and revised the manuscript. DB participated to conception and design, acquisition and interpretation of data, was involved in drafting the manuscript and revised it critically for important intellectual content. All authors have read and approved the final manuscript.

## Acknowledgements

Financial support for this study has been provided by GlaxoSmithKline, Access to Medicines Centre of Excellence, UK.

## Pre-publication history

The pre-publication history for this paper can be accessed here:

http://www.biomedcentral.com/1471-2466/13/15/prepub
